# Occurrence and genetic diversity of CRESS DNA viruses in wild birds: a Hungarian study

**DOI:** 10.1038/s41598-020-63795-x

**Published:** 2020-04-27

**Authors:** Eszter Kaszab, György Lengyel, Szilvia Marton, Ádám Dán, Krisztián Bányai, Enikő Fehér

**Affiliations:** 1 Reorganization of the Hungarian Academy of Sciences, Budapest, Hungary; 2 Hungarian Defence Forces Military Medical Centre, Budapest, Hungary; 3 University of Veterinary Medicine, Budapest, Hungary

**Keywords:** Viral genetics, Virology

## Abstract

Circoviruses, cycloviruses and other circular, replication-associated protein-encoding single stranded (CRESS) DNA viruses have been detected in a variety of animal taxa. In this study, cloacal swab samples (n = 90) were examined for CRESS DNA viruses from 31 wild bird species living at various aquatic sites in Hungary to identify possible reservoirs of viruses pathogenic to domestic poultry. A total of 30 (33.3%) specimens tested positive with pan-CRESS DNA virus specific PCR. Goose circovirus (GoCV), Duck associated cyclovirus 1 (DuACyV-1) and Garrulus glandarius associated circular virus 1 (GgaCV-1) were detected in nine, three and two different bird species, respectively. Selected specimens were subjected to whole genome sequencing. The obtained sequence data revealed conserved gene structure within the identified virus species and detected homologous (within GoCV) and possible heterologous recombination (within DuACyV-1) events. Results presented here provide new information on the genomic diversity and evolution of selected CRESS DNA viruses.

## Introduction

Members of the *Circovirus* (CV) and *Cyclovirus* (CyV) genera (*Circoviridae* family) have circular, single-stranded DNA genomes of 1.7–2.1 kb length enclosed in non-enveloped, icosahedral virions^1^. The replication-associated protein (*rep*) and capsid (*cp*) genes are transcribed bidirectionally^1^.The stem-loop structure at the 5’ intergenic region has a role in the initiation of rolling-circle replication. Binding of the Rep protein near the origin of replication induces a nick in the nonanucleotide sequences of the stem-loop with its helicase and endonuclease activity supplying primer for the rolling circle amplification^1^. Circoviruses and cycloviruses could be distinguished based on the *rep* and *cp* position; the circoviral *rep* is located on the virion strand, while for cycloviruses this gene is encoded on the complementary strand^1,2^. The size of the intergenic region located between the stop codons of the two major ORFs could be also different and may be missing in some cycloviruses^1–5^.

A number of other circular, Rep-encoding single-stranded (CRESS) DNA viruses, which are currently not classified into these two genera, have been also characterized and may differ considerably in the genomic structure and gene orientation; for example, the genes of these viruses are either unidirectionally or bidirectionally oriented and the encoded *rep* and *cp* can be partially overlapping^1,6–8^. With the development and general use of sequencing methods the amount of described CRESS DNA genomes have been rapidly expanded. To facilitate and reconsider the taxonomy of non-circoviral and non-cycloviral genomes, Rosario *et al*. established eight (I-VIII) types of genomes for grouping CRESS DNA viruses and made recommendations for reporting and classifying those^3^.

CRESS DNA viruses have been identified in terrestrial animals and marine organisms as well as environmental specimens collected in freshwater lakes, sewage or soil^1,8–15^. Viruses of the *Circoviridae* family have been detected in domesticated, pet and wild birds. Some of these viruses (e.g., *Beak and feather disease virus*, *Pigeon circovirus*, or *Goose circovirus*) may cause serious infections and marked economical losses^1,16–18^. Furthermore, due to the provoked immunosuppression associated with circovirus infection the host bird may be predisposed to secondary infections by other microbes^16,19^.

In the present study we aimed at investigating the role of wild birds in the maintenance and transmission of pathogenic circoviruses. We applied a pan-circovirus PCR assay combined with sequencing of the amplified gene fragment. Based on the obtained gene sequences we performed whole genome sequencing on a subset of the identified circoviruses and other CRESS DNA viruses. Whole genome sequencing not only confirmed that the amplified gene fragments are parts of circular viral genomes but also shed light on the evolutionary mechanisms of differentiation of the identified CRESS DNA viruses.

## Results

### High positivity rate of CRESS DNA viruses in freshwater habitats

**Figure 1:**
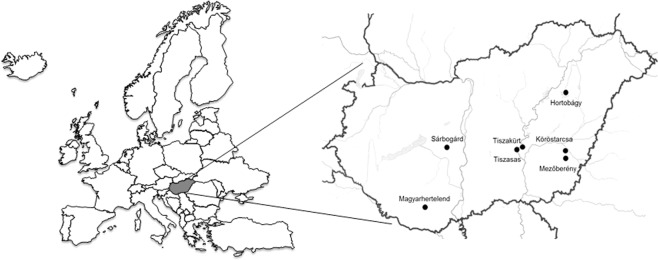
The geographic origin of diagnostic PCR positive cloacal samples examined in this study.

Altogether 30 of the 90 cloacal samples tested positive with the screening PCR assay, representing 12 species of host birds (Table 1, S1). Three groups of sequences were distinguished by direct sequencing and BLASTn search of the nested PCR products, showing similarities with the *rep* of *Goose circovirus* (GoCV, n = 9), *Duck associated cyclovirus 1* (DuACyV-1, n = 17) and Garrulus glandarius associated circular virus 1 (GgaCV-1, n = 2) (Table 1). Two additional PCR amplicons could not be analyzed due to the low resolution sequence chromatograms, likely because these contained a mixture of different *rep* sequences. The GoCV sequences (10% positivity of all samples) originated from variable species of Anseriformes, Suliformes, Pelecaniformes, Passeriformes and Accipitriformes from Sárbogárd (8/31, 25.8%) and Hortobágy (1/29, 3.4%) (Table 1). The hosts of the GgaCV-1 strains were Eurasian jay (*Garrulus glandarius*) and common teal (*Anas crecca*), both sampled near Sárbogárd. The DuACyV-1 strains were collected from birds of Anseriformes and Podicipediformes near Mezőberény (16/17, 94.1%) and Köröstarcsa (1/5, 20.0%). The host birds and collection place of the cloacal swabs are presented in Table 1 and Fig. 1.

**Table 1:** The host species, the collection place and detected viral sequences of diagnostic PCR positive wild bird samples.

Host species	Place of collection	Nested PCR product	Strain/Acc.no.
Garrulus glandarius (Eurasian jay)	Sárbogárd, Fejér county, Hungary	GgaCV-1	GgaCV-1/1/ KY884302
Anas crecca (common teal or Eurasian teal)		GgaCV-1	GgaCV-1/2/ MG254877
Anas platyrhynchos (mallard)		GoCV	GoCV-Hun1/ MG254878
Anser anser (greylag goose)		GoCV	GoCV-Hun2/ MG254879
Anser anser (greylag goose)		GoCV	—
Anser albifrons (greater white-fronted goose)		GoCV	GoCV-Hun3/ MG254880
Phalacrocorax carbo (cormorant)		GoCV	—
Ardea cinerea (grey heron)		GoCV	—
Buteo buteo (common buzzard)		GoCV	—
Pica pica (common or European magpie)		GoCV	—
Corvus frugilegus (rook)		Low resolution	—
Ciconia ciconia (white stork)	Hortobágy, Hajdú-Bihar county, Hungary	Low resolution	—
Haliaeetus albicilla (erne or Eurasian sea eagle)		GoCV	—
Anas platyrhynchos (mallard)	Mezőberény, Békés county, Hungary	DuACyV	DuACyV-1/1/ KY851116
Anas platyrhynchos (mallard)		DuACyV	DuACyV-1/3/ MG254874
Anas platyrhynchos (mallard)		DuACyV	—
Anas platyrhynchos (mallard)		DuACyV	—
Anas platyrhynchos (mallard)		DuACyV	—
Anas platyrhynchos (mallard)		DuACyV	—
Anas platyrhynchos (mallard)		DuACyV	—
Podiceps cristatus (great crested grebe)		DuACyV	DuACyV-1/2/ MG254873
Podiceps cristatus (great crested grebe)		DuACyV	DuACyV-1/5/ MG254876
Anser erythropus (lesser white-fronted goose)		DuACyV	DuACyV-1/4/ MG254875
Anser erythropus (lesser white-fronted goose)		DuACyV	DuACyV-1/4/ MG254875
Anser erythropus (lesser white-fronted goose)		DuACyV	—
Anser erythropus (lesser white-fronted goose)		DuACyV	—
Anser erythropus (lesser white-fronted goose)		DuACyV	—
Anser erythropus (lesser white-fronted goose)		DuACyV	—
Anser erythropus (lesser white-fronted goose)		DuACyV	—
Anser erythropus (lesser white-fronted goose)	Köröstarcsa, Békés county, Hungary	DuACyV	—

### Genomic diversity of CRESS DNA viruses

**Figure 2:**
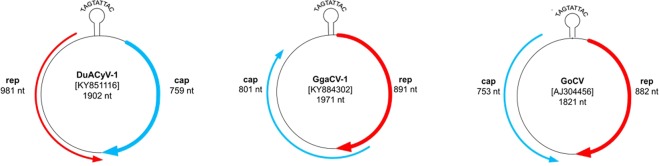
Genomic organization of the *Duck associated cyclovirus 1* (DuACyV-1), the Garrulus glandarius associated circular virus 1 (GgaCV-1) and *Goose circovirus* (GoCV).

Because genomic fragments of CRESS DNA viruses have been commonly found in eukaryotic genomes as a consequence of ancient DNA integration events, it was important to verify that the amplified partial *rep* genes are parts of circular viral genomes^20^. Therefore, we performed whole genome amplification and sequencing. Back-to-back PCR amplicons were generated for nine specimens not analyzed earlier^6,21^ and then subjected to next generation sequencing (Table 1). Five, three and one sequences showed similarities with the genome of DuACyV-1, GoCV, and GgaCV-1, respectively. The *rep* and *cp* genes, and control motifs typical for the reference genomes were readily identified in all viral genomes (Fig. 2).

**Figure 3:**
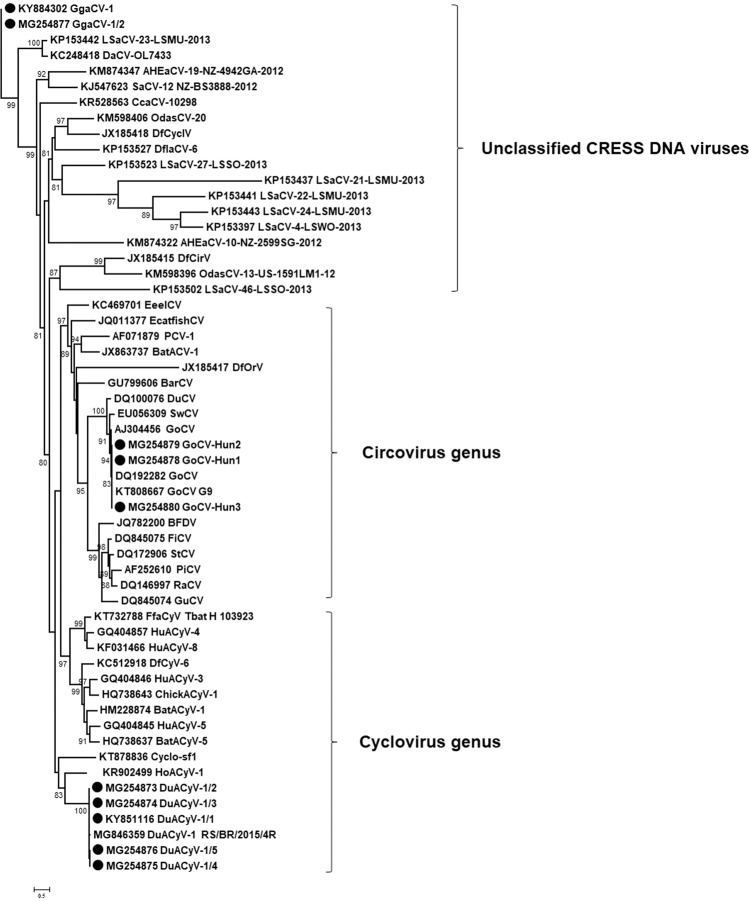
Maximum likelihood phylogenetic tree of representative Rep aa sequences. Sequences identified in this study were highlighted by dots. The tree was generated by the PhyML online software with the best fitted LG + G + I model. SH-like support values <80 were not shown. Substitution per site is represented by the scale bar.

**Figure 4:**
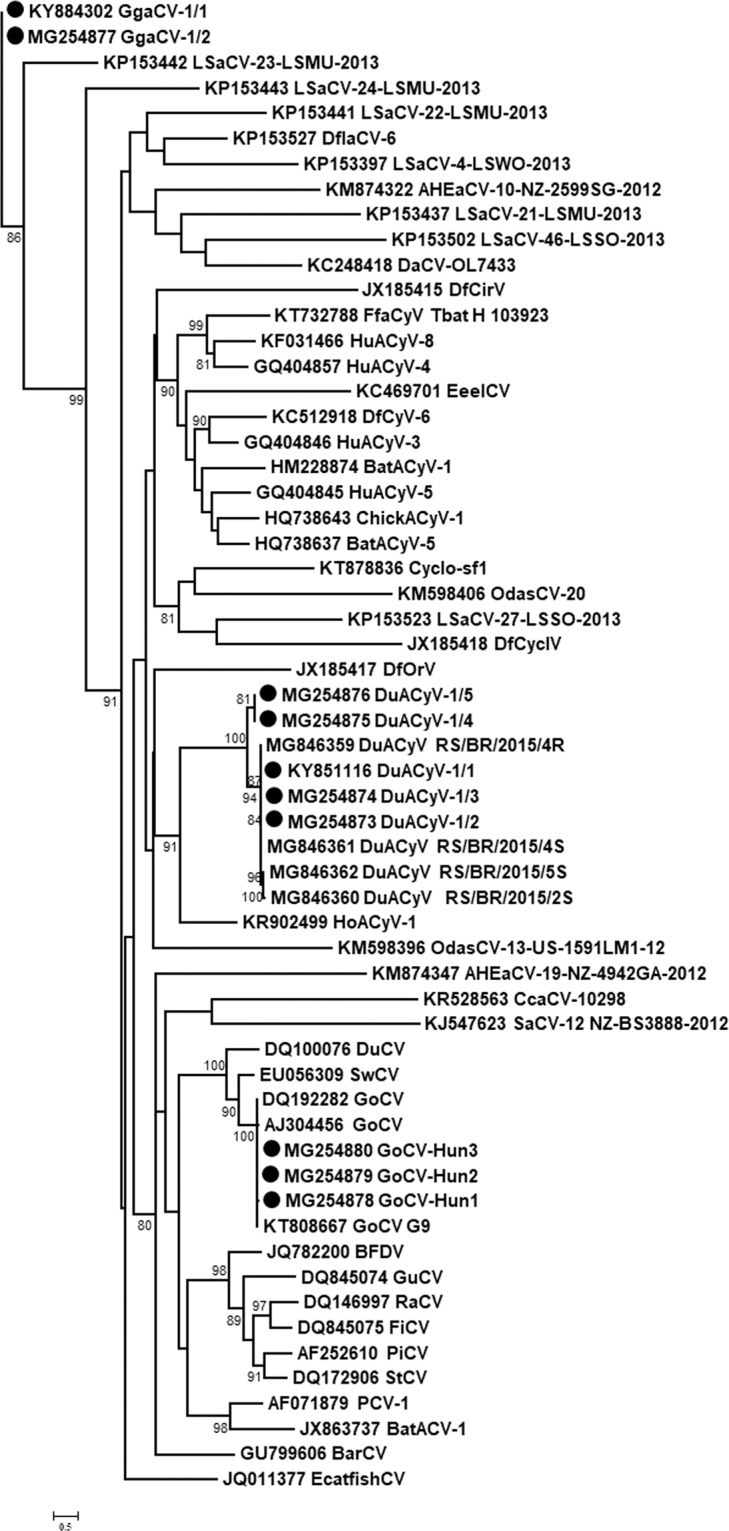
Maximum likelihood phylogenetic tree of representative Cp aa sequences. Sequences identified in this study were highlighted by dots. The tree was generated by the PhyML online software with the best fitted RtREV + G + F model. SH-like support values <80 were not shown. Substitution per site is represented by the scale bar.

**Figure 5:**
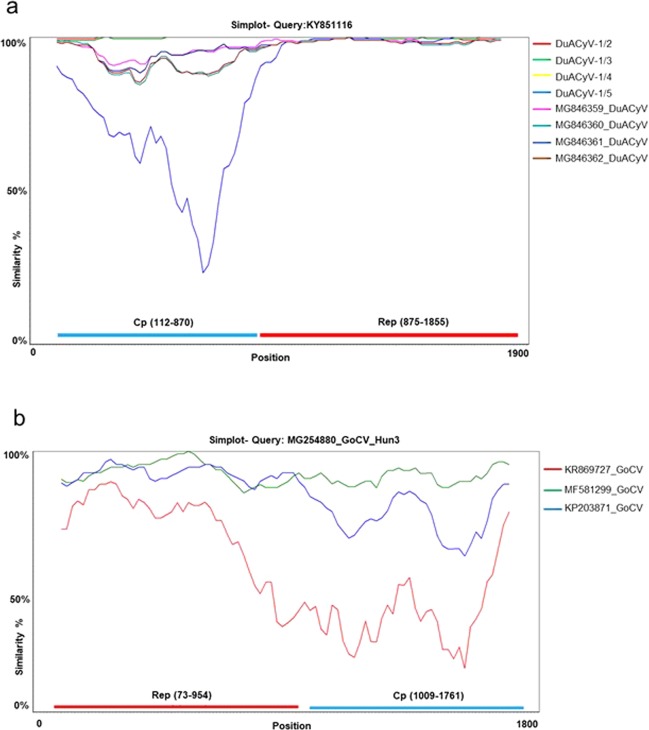
Recombination analysis of *Duck associated cyclovirus 1* or DuACyV (**a**) and *Goose circovirus* or GoCV (**b**) complete genome sequences using Similarity plot.

The DuACyV-1 genomes encoded *cp* on the viral strand and *rep* on the complementary strand, with a 158 nt long non-coding region (NCR) between the 5’ ends and a four nt long NCR between the 3’ ends of the two genes. The nonanucleotide motif sequence in the NCR upstream of the *cp* was TAGTATTAC for all DuACyV-1 genomes^21^ (Fig. 2). The 1902 nt long genomes of three strains (one sequence from great crested grebe or *Podiceps cristatus* and two sequences from mallards or *Anas platyrhynchos*) were near identical (99.7–99.8% genome-wide identity) with the same length of the *rep* and *cp*. Three newly determined DuACyV-1 genomes with a length of 1899 nt (two sequences from lesser white-fronted goose or *Anser erythropus* and one sequence from great crested grebe) were fully identical in their sequences (Fig. 3 and Fig. 4). The *cp* genes derived from these shorter viral genomes were characterized by a three nt (i.e. one aa) deletion in the 5’ region when compared with the *cp* genes of the variants with longer genome. The nt and aa identities of the Rep of strains DuACyV-1/1–1/5 (Table 1) ranged between 99.2–100% and 98.8–100%, while those for Cp it ranged between 72.1–100% and 73.4–100%, respectively. The genome wide identity was 88.6–100% for Hungarian DuACyV strains. DuACyV-1/1–1/5 showed 87.4–100% identity with viral sequences derived from fecal samples of diarrheic chickens (*Gallus gallus*) from Brazil^22^ (Fig. 3 and Fig. 4). In the phylogenetic tree of the Cp (Fig. 4) the strain DuACyV-1/4 and 1/5 grouped separately from the other known DuACyV sequences. On the other hand, in case of Rep, all DuACyV sequences clustered in a common branch (Fig. 3). To clarify this discrepancy, recombination analyses were carried out using the complete genomic sequences. Based on the RDP4 (with an average p-value between 2,023 ×10^−02^ and 1.230 ×10^–23^ as estimated by the different methods) and SIMPLOT analysis, putative recombination events were detected within the *cp* gene (Fig. 5). Unfortunately, due to the limited number of DuACyV sequences available currently in GenBank, it was not possible to infer the precise phylogenetic relationships and possible origins of the divergent capsid-coding regions within the DuACyV genomes.

**Figure 6:**
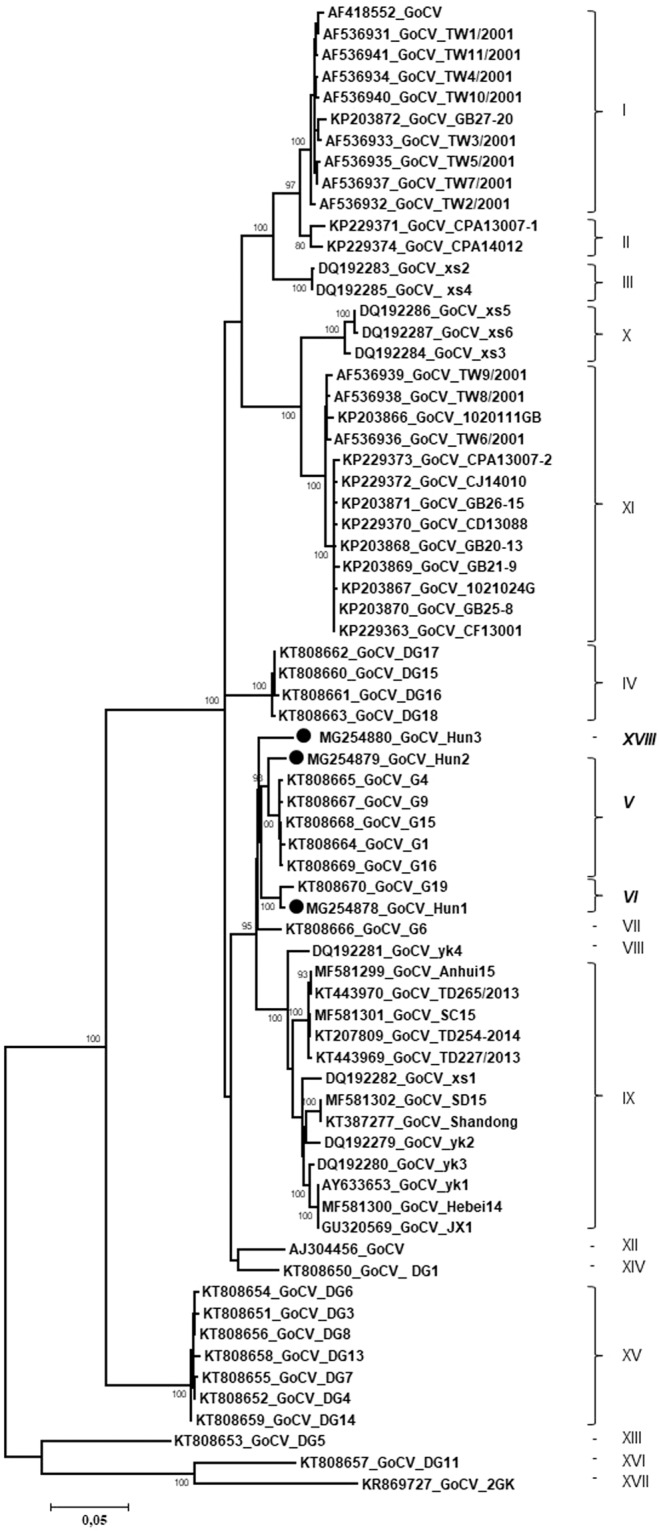
Maximum likelihood phylogenetic tree of goose circovirus complete genome sequences. Sequences identified in this study were highlighted by dots. The tree was generated by the PhyML online software with the best fitted TN93 + G + I model. SH-like support values <80 were not shown. Substitution per site is represented by the scale bar.

The 1822 nt long GoCV genomes (n = 3) were characterized from cloacal swabs of wild mallard, wild greylag goose (*Anser anser*) and greater white-fronted goose (*Anser albifrons*). The GoCV genomes encoded an 882 nt long (nt 73–954) *rep* on the viral strand and a 783 nt long (nt 1791–1009) *cp* on the complementary strand with a 104 nt long NCR between the 5’ ends, and a 54 nt long NCR between the 3’ ends of the two major genes (Fig. 2). The nt and aa identities of the *rep* ranged between 97.3–97.7% and 99.3–99.8%, while those for the *cap* fell between 95.6–96.8% and 97.3–98.8%, respectively (Fig. 3 and Fig. 4). The Hungarian genomic sequences showed 96.8–97.3% genome-wide identities. The Hungarian strains (strain GoCV-Hun1, -Hun2 and -Hun3) shared 97.0–99.4% genome-wide identities with the most closely related GenBank reference sequences collected from wild birds in Poland (Fig. 5)^19^. Recombination analysis using the RDP4 (with SiScan method, average p-value of 9.664 ×10^–08^) and SIMPLOT software detected a common potential recombination event affecting the genomes of all three Hungarian GoCV strains and sequences from Poland (Fig. 5). In the phylogenetic analysis (Fig. 6), the Hun1 strain clustered together with a genotype VI strain (detected in wild greylag goose), while the Hun2 strain grouped with genotype V GoCV sequences. The strain Hun3 with a maximum of 96.9–97.1% whole genome sequence identity value was distinct from both genotype V and VI GoCVs and likely represents a novel genotype that we tentatively called genotype XVIII^19^.

The 1971 nt long genomes of the GgaCV-1 strains were identical (Figs. 3–4). The sequences were derived from distantly related bird species, the Eurasian jay and common teal (Table 1)^6^. These genomes coded for unidirectionally located, overlapping 891 nt long *rep* and 801 nt long *cp* on the viral strand with a 335 nt long NCR between the 5’ end of the *rep* and 3’ end of the *cp*, which encompassed the circovirus-like nonanucleotide motif of TAGTATTAC^6^ (Fig. 2). Phylogenetic analyses placed both virus genomes in basal position when compared to CyVs and CVs.

## Discussion

In this report we studied the occurrence of CRESS DNA viruses in cloacal samples of wild birds. Then we performed whole genomic analyses that permitted further insight into the genomic structure and evolutionary mechanisms of the identified viruses. A novel CRESS DNA virus, GgaCV-1, was uniquely identified in Hungary in two distantly related avian species, Eurasian teal and Eurasian jay^6^. Another newly described virus, DuACyV-1, since its first report^21^ has been described in other waterfowls in Hungary and chickens in Brazil^22^. The question whether these two CRESS DNA viruses are pathogenic or innocuous viruses is open. A third group of CRESS DNA viruses, the GoCV, which is pathogenic to domestic geese, also shows a wider host spectrum than previously thought. Among bird species other than Anseriformes, GoCVs were detected in cormorant (*Phalacrocorax carbo*), grey heron (*Ardea cinerea*), European magpie (*Pica pica*), common buzzard (*Buteo buteo*) and Eurasian sea eagle (*Haliaeetus albicilla*) (Table 1). Furthermore, during the preparation of this manuscript, partial GoCV *cp* sequences became available in the DNA databases (unpublished GenBank records); these sequences derived from intestinal or other internal organ (liver, heart, kidney, gizzard) specimens of wild birds (including wild greylag goose, mallard, white stork (*Ciconia ciconia*), western jackdaw (*Corvus monedula*), European herring gull (*Larus argentatus*), brambling (*Fringilla montifringilla*), and rock dove (*Columba livia*)) collected in Poland between 2014 and 2017. Detection of GoCV in the internal organs may indicate active virus replication in distantly related wild bird species. This finding suggests that numerous wild bird species may serve as natural reservoirs for GoCV facilitating virus transmission among flocks of domestic geese. While analyzing the geographical data we observed that some CRESS DNA virus species were more common in a particular study area than in others (e.g., GoCV in Sárbogárd, DuACyV-1 in Mezőberény) raising the possibility that as yet unidentified factors may be more important in shaping the ecology and epizootiology of (potentially) pathogenic CRESS DNA viruses than the taxonomy, behavior or lifestyle of affected bird species. Nonetheless, an analytical evaluation of possible host species association with viral shedding was prevented by the low number of samples available for this study and by different sampling pattern of bird species at various study sites. Despite limitations that arose from the relatively low number of samples, our study provided new data on geographical distribution and genetic diversity of the identified CRESS DNA viruses.

Wild birds, especially waterfowls, are able to carry low or high pathogenic avian viruses which can spread to geographically distant regions via their migration routes^23^. Influenza virus well exemplifies how long-distance migration birds contribute to the dispersal of highly pathogenic virus strains around the circumpolar region and help the virus to become globally spread within short period^23^. Hungary is a nesting area and a preferred resting place for wild greylag goose, greater white-fronted goose and mallard^24^. They migrate from North Europe to Middle and East Europe, and from the Carpathian Basin to South Europe and North Africa^24^. CRESS DNA viruses may be present in freshwater ecosystems and may have a role in virus circulation among distant freshwater habitats. This possibility on long-distance dispersal has now been demonstrated for GoCV and for DuACyV-1. Thus, for example, genetically closely related GoCVs were detected in Poland and Hungary. Additionally, DuACyV-1-like partial *rep* sequence was identified in human stool sample in Tunisia (GenBank acc.no. GQ404902) and DuACyV-1 genomes were detected in diarrheal chicken in Brazil^22^, suggesting a worldwide distribution of this virus species.

By analyzing the whole virus genomes from our sample archival we observed the following features with regard to the genetic variation of CRESS DNA viruses. (i) The genomic organization of the identified GoCVs, DuACyVs and GgaCVs strains was classified into CRESS DNA virus genome type 1, type 2 and type 5, respectively^3^. The main criteria that permit the classification into CRESS DNA virus genome types are the polarity and orientation of *cp* and *rep* genes, and the number of genes encoded by the genome^1,3,6,21^. (ii) Accumulation of point mutations resulted in limited genetic diversity among co-circulating CRESS DNA virus strains. This holds true even for GoCVs, which can be classified into XVII genotypes and one additional genotype described in the present study^19^. The three genotypes of GoCV were detected in the same Hungarian geographic area but from distinct host species. Similar findings were reported from Poland^19^. Despite the genetic diversity of GoCVs, low non-synonymous to synonymous substitution ratio was calculated (dN/dS, 0.0581 and 0.103 for the *rep* and *cp*, respectively; data not shown), implying that chiefly purifying selection acts on the genes of GoCVs. It needs to be monitored whether accumulation of mutations may help virus adaptation to novel hosts and could contribute to the selection of various genotypes in these novel host species over a long time. (iii) Recombination was found to shape the genomic evolution of the pathogenic GoCV as well as the DuACyV strains^19^. The recombination seen in GoCVs involved homologous viruses. In contrast, the parental strain that donated the cp gene for the recombinant DuACyVs is currently unknown and it is possible that the recombination event seen in these DuACyVs occurred between homologous or heterologous virus species. The relatively low *cp* gene sequence identity (nt, 72.1–72.4%; aa, 73.4–73.8%) in the two major variants of DuACyV might suggest that the recombination occurred between viruses belonging to different CyV species. Similar inter-virus-species recombination events were reported for another group of CyVs, the dragonfly origin DfCyVs^25^. This possibility is further corroborated in the recent revision of CRESS DNA virus taxonomy where the authors claim that the 80% identity threshold established to demarcate distinct CV and CyV species generally holds true for pairwise comparisons of either the *cp* or *rep* gene sequences^1^.

In summary, our virus survey provides evidence of high detection rate of CRESS DNA viruses in wild bird samples around Hungarian lakes. More structured sampling that includes additional hosts and environmental specimens and extends the number of sites and duration of sample collection could help better understand the ecology and epizootiology of potentially pathogenic CRESS DNA viruses. New genome sequence data indicate that recombination among homologous and even among heterologous viral genomes contributes to the genetic diversity of CRESS DNA viruses highlighting the need for whole genome sequencing to become a routine approach when analyzing genetic diversity during virus surveillance studies.

## Materials and methods

### Samples

General considerations: Cloacal swabs and fecal samples were thought to serve as suitable materials to explore the diversity of (potentially) pathogenic CRESS DNA viruses^1^. The cloacal swabs were collected at variable country sites by veterinarians, hunters and ornithologists, who volunteered to contribute to the work of the national avian influenza virus surveillance network in Hungary. In terms of numbers and species of birds, sample collection was a fairly random process. Neither the minimum nor the maximum numbers of samples to be collected were defined. Consequently, at some study sites the prevailing wild bird species were sampled, whereas at other study sites the sampling better reflected the local bird species diversity. This random sampling resulted in differences in the host species composition of sample collections that prevented us from performing host species specific analysis of data.

The 90 cloacal swab specimens from 31 wild bird species were collected for compulsory seasonal influenza virus surveillance in November and December, 2013, at different lakes in Hungary near Sárbogárd (n = 31), Hortobágy (n = 29), Mezőberény (n = 17), Köröstarcsa (n = 5), Tiszakürt (n = 1), Tiszasas (n = 4), Magyarhertelend (n = 3)^6,21^. The samples were sent for influenza testing to the Veterinary Diagnostic Directorate, National Food Chain Safety Office, Budapest, Hungary. In this study we processed the leftover sample materials, provided by the Veterinary Diagnostic Directorate, for CRESS DNA virus detection. Data about the age, gender or health status (alive or dead) of the hosts were not noted in each case.

### DNA extraction and amplification methods

The cloacal swab specimens were eluted in 1 ml of PBS buffer. Nucleic acid was extracted with Direct-zol RNA MiniPrep Kit (Zymo Research) according to the manufacturer’s instruction but omitting the DNase treatment. The primer sets designed by Li *et al*^2^. were used for CV and CyV PCR amplifying an approximately 400 bp long fragment of the *rep* gene^2,6,20,21^. The 25 μl reaction volume contained 1 μl of the extracted nucleic acid, 200 nM of primers, 200 μM of dNTP mix, 1x DreamTaq Green buffer and 0.625 U of DreamTaq DNA Polymerase (Thermo Fisher Scientific). The first and second round PCRs contained the step of initial denaturation at 95 °C for 3 min, 40 cycles of denaturation at 95 °C for 30 sec, primer annealing at 52 °C (first round of nested PCR) and 56 °C (second round of nested PCR) for 30 sec and extension at 72 °C for 1 min, followed by a final extension step at 72 °C for 10 min^6,20,21^. The nested PCR products were cut and purified from agarose gel by the Geneaid Gel/PCR DNA Fragments Extraction Kit and sequenced with the usage of BigDye Terminator v1.1 Cycle Sequencing Kit (Thermo Fisher Scientific) and ABI PRISM 3100-Avant Genetic Analyzer.

Viral circular complete genomes were amplified by back-to-back PCR primers fitting, respectively, to the sequences of the nested PCR products. The 25 μl reaction volume contained 1 μl of the extracted nucleic acid, 200 nM of primers, 200 μM of dNTP mix, 1x Phusion Green HF buffer and 0.25 U of Phusion DNA Polymerase (Thermo Fisher Scientific). The back-to-back PCRs contained the steps of initial denaturation at 98 °C for 30 sec, 45 cycles of denaturation at 98 °C for 10 sec, annealing at 60–61 °C (depending of the primer set) for 30 sec and extension at 72 °C for 1 min, followed by a final extension step at 72 °C for 10 min^6,20,21^. The PCR products were purified with Geneaid Gel/PCR DNA Fragments Extraction Kit.

### Genome sequencing

Next generation sequencing was carried out with the Ion Torrent Personal Genome Machine™ (PGM) System. Enzymatic fragmentation and adapter ligation of the back-to-back PCR products was carried out with NEBNext® Fast DNA Fragmentation & Library Prep Set for Ion Torrent™ (New England Biolabs), while the fragments were barcoded with the Ion Xpress™ Barcode Adapters (Thermo Fisher Scientific). The barcoded samples were purified with Geneaid Gel/PCR DNA Fragments Extraction Kit, separated on a 2% precast gel (Thermo Fisher Scientific). Products between 300–350 bp were amplified with the PCR mixture of the NEBNext® Fast DNA Fragmentation & Library Prep Set for Ion Torrent^TM^ kit (NEB). The amplification protocol steps were initial denaturation at 98 °C for 30 sec, followed by 12 amplification cycles at 98 °C for 10 sec, 58 °C for 30 sec and 72 °C for 30 sec, and termination at 72 °C for 5 min. The amplified library DNA was extracted from gel and was quantified with Qubit® 2.0 Fluorometer using Qubit™ dsDNA BR Assay Kit (Thermo Fisher Scientific). Barcoded products were mixed and processed in emulsion PCR according to the manufacturer’s protocol using an Ion PGM™ Template Kit on an OneTouch™ v2 instrument. Templated bead enrichment on an Ion OneTouch™ ES machine and further steps were performed according to the 200 bp sequencing protocol (Thermo Fisher Scientific). The Ion PGM™ Sequencing Kit on a 316 chip was used for sequencing.

### Genome analysis and genomic recombination

Raw sequence data were mapped to references applying the Geneious software^26^. The sequence and structure of some novel genomes were confirmed by primer walking method from inverse PCR products. The complete genomic sequence of a novel cyclovirus species (DuACyV-1, GenBank acc. no. KY851116) and another CRESS DNA virus (GgaCV-1, GenBank acc. no. KY884302) were reported from our laboratory ^6,21^. Whole genome sequences determined in this study were deposited in the GenBank with accession numbers MG254873-MG254880.

The initial sequence analysis included the use of online analysis tools, BLAST (https://blast.ncbi.nlm.nih.gov/Blast.cgi) and ORF Finder (https://www.ncbi.nlm.nih.gov/orffinder/). The muscle algorithm of the AliView and MEGA6 software was used for making alignments of nt and aa sequences^27,28^. Maximum likelihood phylogenetic trees of the Rep and Cp aa sequences and the GoCV complete genome sequences were generated using the PhyML online software with the best fitted models and were visualized with the MEGA6 software (shown in figure legends)^28,29^.

Possible recombination events were detected with the RDP, GeneConv, Bootscan, MaxChi, Chimaera, SiScan and 3Seq recombination detection methods embedded in the Recombination Detection Program (RDP4) v4.97^30^. Bonferroni correction was used as a default setting. Similarity plot analysis was executed in SIMPLOT software package v3.2^31^. The analysis was performed under the Kimura-2 parameter (K2P) model with a window size of 200 bp, step size of 20 bp and a transition/transversion ratio of 2.

The selection constraint and the dN/dS ratio were calculated with the fixed effect likelihood (FEL), the fast unconstrained bayesian approximation for inferring selection (FUBAR), the single-likelihood ancestor counting (SLAC) and the mixed effects model of evolution (MEME) methods of the Datamonkey server^32^.

### Ethical approval

This article does not contain any studies with animals performed by any of the authors.

## Supplementary information

Supplementary table S1.

## Data Availability

The sequence data supporting this study are available in the GenBank with the accession numbers MG254873-MG254880.
