# Publisher Correction: Occurrence and genetic diversity of CRESS DNA viruses in wild birds: a Hungarian study

**DOI:** 10.1038/s41598-020-67585-3

**Published:** 2020-06-22

**Authors:** Eszter Kaszab, György Lengyel, Szilvia Marton, Ádám Dán, Krisztián Bányai, Enikő Fehér

**Affiliations:** 1 Institute for Veterinary Medical Research, Centre for Agricultural Research, Budapest, Hungary; 2 Hungarian Defence Forces Military Medical Centre, Budapest, Hungary; 3 University of Veterinary Medicine, Budapest, Hungary

Correction to: *Scientific Reports* 10.1038/s41598-020-63795-x, published online 27 April 2020

The original version of this Article contained an error in Affiliation 1, which was incorrectly given as ‘Reorganization of the Hungarian Academy of Sciences, Budapest, Hungary’. The correct affiliation is listed below:

Institute for Veterinary Medical Research, Centre for Agricultural Research, Budapest, Hungary.

