# Supplementary material for: Occurrence and genetic diversity of CRESS DNA viruses in wild birds: a Hungarian study
Source: Sci Rep. 2020 Apr 27;10:7036. doi: 10.1038/s41598-020-63795-x (PMC7184566; doi:10.1038/s41598-020-63795-x)
Supplement: Supplementary file 1 — Supplementary table S1. [file 41598_2020_63795_MOESM1_ESM.pdf]

Occurrence and genetic diversity of CRESS DNA viruses in wild birds: a Hungarian study

Eszter Kaszab[1], György Lengyel[2], Szilvia Marton[1], Ádám Dán[3], Krisztián Bányai[1+], Enikő Fehér[1+*]

[1] Institute for Veterinary Medical Research, Centre for Agricultural Research, Budapest, Hungary

[2] Hungarian Defence Forces Military Medical Centre, Budapest, Hungary

[3] University of Veterinary Medicine, Budapest, Hungary

+ These authors contributed equally to this work

* feher.eniko@agrar.mta.hu

Supplementary table S1. The host species, site of collection, circovirus/cyclovirus screening PCR positivity and Sanger sequencing results of the samples processed in this study.

Host	Place of collection	PCR positive sample no. /investigated sample no.	Sequencing result
Anas platyrhynchos (mallard)	Sárbogárd	1/6	GoCV
	Mezőberény	7/7	DuACyV-1
	Magyarhertelend	0/3	-
	Köröstarcsa	0/3	-
	Tiszasas	0/2	-
Anser anser (greylag goose)	Sárbogárd	2/2	GoCV
Anser albifrons (greater white-fronted goose)	Sárbogárd	1/3	GoCV
Ardea cinerea (grey heron)	Sárbogárd	1/2	GoCV
Buteo buteo (common buzzard)	Sárbogárd	1/1	GoCV
	Hortobágy	0/3	-
Phalacrocorax carbo (cormorant)	Sárbogárd	1/2	GoCV
Pica pica (common or European magpie)	Sárbogárd	1/1	GoCV
Garrulus glandarius (Eurasian jay)	Sárbogárd	1/1	GgaCV-1
Anas crecca (common teal or Eurasian teal)	Sárbogárd	1/1	GgaCV-1
Corvus frugilegus (rook)	Sárbogárd	1/3	Low resolution
Anser fabalis (bean goose)	Sárbogárd	0/1	-
Chroicocephalus ridibundus (black-headed gull)	Sárbogárd	0/1	-
Fulica atra (eurasian coot)	Sárbogárd	0/3	-
Gallinula chloropus (common moorhen)	Sárbogárd	0/1	-
Phasianus colchicus (common pheasant)	Sárbogárd	0/1	-
Streptopelia decaocto (Eurasian collared dove)	Sárbogárd	0/1	-
	Tiszakürt	0/1	-
Asio otus (long-eared owl)	Sárbogárd	0/1	-
	Hortobágy	0/2	-
Haliaeetus albicilla (erne or Eurasian sea eagle)	Hortobágy	1/4	GoCV
Ciconia ciconia (white stork)	Hortobágy	1/3	Low resolution
Pandion haliaetus (osprey)	Hortobágy	0/1	-
Accipiter nisus (Eurasian sparrowhawk)	Hortobágy	0/1	-
Falco tinnunculus (common kestrel)	Hortobágy	0/2	-
Circus aeruginosus (western marsh-harrier)	Hortobágy	0/3	-
Falco subbuteo (Eurasian hobby)	Hortobágy	0/3	-
Nycticorax nycticorax (black-crowned night-heron)	Hortobágy	0/1	-
Tyto alba (barn owl)	Hortobágy	0/3	-
Corvus corax (common raven)	Hortobágy	0/2	-
Athene noctua (little owl)	Hortobágy	0/1	-
Passer domesticus (house sparrow)	Tiszasas	0/2	-
Podiceps cristatus (great crested grebe)	Mezőberény	2/2	DuACyV-1
Anser erythropus (lesser white-fronted goose)	Mezőberény	7/8	DuACyV-1
	Köröstarcsa	1/2	DuACyV-1
